# Molecular Dynamics Study on the Compressive Behavior of Intermetallic Compounds in 3xxx Aluminum Alloys

**DOI:** 10.3390/ma19030535

**Published:** 2026-01-29

**Authors:** Yexin Li, Jingyuan Bai, Zhou Yang, Zhongjie Chen, Chuanyang Wang, Quanfeng Zheng, Di Tie

**Affiliations:** 1 School of Intelligent Manufacturing, Lishui Vocational and Technical College, Lishui 323000, China; yangzhou5959@163.com; 2 College of Mechanical Engineering, Shenyang University, Shenyang 110044, China; liyexin0902@163.com; 3 Suzhou Jiangjin Automation Technology Co., Ltd., Suzhou 214122, China; zhongjie.chen@auto-jiangjin.com; 4 School of Mechanical & Electric Engineering, Soochow University, Suzhou 215006, China; cywang@suda.edu.cn; 5 Zhejiang Xiafeng Precision Die-Casting Co., Ltd., Lishui 323900, China; quanfeng2026@163.com; 6 School of Materials Science and Engineering, Guangdong Ocean University, Zhanjiang 529500, China

**Keywords:** intermetallic compounds, mechanical properties, molecular dynamics, 3xxx alloy

## Abstract

The morphology and distribution of intermetallic compounds (IMCs), such as Al_6_Mn, Al_2_Cu, and Al_12_Fe_3_Si_2_, play a critical role in determining the mechanical properties of 3xxx series aluminum alloys. In this study, the compressive behavior of these IMCs was systematically investigated using the modified embedded atom method (MEAM) potential and the large-scale atomic/molecular massively parallel simulator (LAMMPS) under various temperatures and strain rates. The results show that as the temperature increases from 623 K to 823 K, both the compressive strength and elastic modulus of the IMCs decrease significantly. Al_12_Fe_3_Si_2_ exhibits the lowest compressive strength, ranging from 1.1 to 9.8 GPa, while Al_2_Cu demonstrates the highest compressive strength, ranging from 3.9 to 19.8 GPa. Within this temperature range, Al_6_Mn and Al_3_Fe show relatively poor stability. At a strain rate of 1 × 10^10^ s^−1^, the thermal sensitivity coefficients for compressive strength are 0.010 and 0.008, and those for elastic modulus are 0.173 and 0.126, respectively. In contrast, Al_2_Cu exhibits the best stability, with thermal sensitivity coefficients of 0.005 for compressive strength and 0.041 for elastic modulus. Furthermore, the influence of strain rate diminishes notably under lower temperatures. Across the entire temperature range, Al_2_Cu displays the highest overall stability, with a strain rate sensitivity index ranging from 0.3527 to 0.3738.

## 1. Introduction

The 3xxx aluminum alloy is widely used in the fields of decorative materials, heat exchange materials, photosensitive materials, and welding materials due to the characteristics of low density, high corrosion resistance, good thermal and electrical conductivity, as well as good reflectivity, non-magnetism, excellent mechanical properties, and formability [1,2,3]. However, various alloying elements cannot be fully dissolved in the matrix due to their limited solubility during the solidification process, inevitably forming various intermetallic compounds (IMCs), such as Al_6_Mn, Al_3_Fe, Al_2_Cu, Al_8_Fe_2_Si, and Al_12_Fe_3_Si_2_, on the surface and inside of the alloy [4]. Fine and spherical IMCs with uniform distribution are beneficial to the mechanical property enhancement of the alloy [5,6,7,8]. On the contrary, IMCs with excessive size and high brittleness in the alloy tend to form stress concentration in the alloy, resulting in the sources of crack initiation and propagation [9,10]. Therefore, the factor of IMCs on the alloy has attracted much attention. IMCs undergo higher temperatures and strain rates during the hot pressing process, leading to adverse effects on the mechanical properties of the alloy. Due to the nanoscale size of IMCs and the extremely high strain rate requirements, the experimental instruments for testing IMCs in the laboratory have inherent limitations. Therefore, first-principles calculations based on density functional theory (DFT), molecular dynamics (MD) simulation, and Monte Carlo simulation constitute established methodologies for investigating the mechanical properties of IMCs. This study adopts the MD simulation method, and the temperature dependence of IMCs has been studied previously [11,12,13,14].

Few studies on the IMCs of the mechanical properties of 3xxx alloys have been reported. Yang et al. [15] reported that the mechanical properties of 3xxx alloy were affected by IMCs at cryogenic temperatures. Based on experiments and simulations, various mechanical properties of IMCs have been evaluated. Fang et al. [16] investigated the microstructure, mechanical, and thermophysical properties of Al_6_Mn, Al_2_Cu, and Al_3_Fe IMCs. The results showed that the compressive properties of the above IMCs were little affected by the three directions during compression, and Al_2_Cu possessed the best isotropy. At 0 K, Al_3_Fe had a high compressive resistance. With the increment of temperature, the degree of thermal mismatch between Al_2_Cu and α-Al matrix declined, and the thermal shock resistance improved. Therefore, Al_2_Cu displayed the best effect in alleviating the initiation and propagation of thermal cracks at the interface. Chen et al. [17] revealed the creep effect of Al_2_Cu through the heating stage using hot-stage nanoindentation technology, of which the hardness was closely related to the creep rate. Tomo et al. [18] also measured the hardness values of Al_12_Fe_3_Si_2_ and Al_3_Fe using nanoindentation.

This study aims to investigate the stress–strain behavior of single-crystal Al_6_Mn, Al_2_Cu, Al_12_Fe_3_Si_2_, Al_8_Fe_2_Si, and Al_3_Fe IMCs under different temperatures and strain rates. The research object is a structural system containing massive atomic unit cells at the nanoscale. Since first-principles calculations are difficult to handle large atomic systems and there are challenges in the experimental characterization of nanoscale samples, this study proposes the Modified Embedded Atomic Method (MEAM) within the MD model to provide a novel strategy for addressing the aforementioned issues.

Using the MD simulation method, the uniaxial stress–strain relationship and deformation evolution of IMCs were investigated at the temperature range from 623 K to 823 K (with an interval of 50 K) and under different strain rates. Additionally, the relationships between ultimate compressive strength and elastic modulus with temperature and strain rate are elucidated. The strain rate sensitivity is calculated based on the Backofen equation [19]. The reliability of this work is confirmed by comparing the MD simulation results with the reported theoretical models, the generally accepted simulation parameter standards, and the benchmark data of similar systems, and by verifying the consistency of key physical quantities during the simulation process.

## 2. Methodology

IMCs (Al_6_Mn, Al_3_Fe, Al_2_Cu, Al_8_Fe_2_Si, and Al_12_Fe_3_Si_2_) in 3003 alloy (a representative 3xxx alloy) underwent compressive deformation along the 1-1 direction (i.e., z-axis direction) with a deformation amount of 0.5, within a temperature range of 623 K to 823 K at 50 K increments, under strain rates of 1 × 10^10^ s^−1^, 5 × 10^10^ s^−1^, 1 × 10^11^ s^−1^, and 5 × 10^11^ s^−1^, respectively. In this study, the LAMMPS software (version number: 29 August 2024) is used to simulate the compressive characteristics of the mentioned IMCs. The single-crystal structure models of these IMCs are constructed by collecting lattice parameters and internal atomic coordinate data. The relevant structure modeling work is completed on the Materials Studio 2023 (MS) platform, and the results are presented in 3D using the Open Visualization Tool (OVITO Basic 3.14.1.) visualization software.

### 2.1. Interatomic Potential

The MEAM exhibits exceptional accuracy in describing intermetallic compound (IMC) atoms when used to characterize interatomic bonding forces. The general equations representing energy are Equations (1) and (2) [20]:

(1) $$E_{total}=\sum_{i}\left(F_{i}({\bar{\rho }}_{i})+\frac{1}{2}\sum_{j\neq 1}S_{ij}\varphi \left(R_{ij}\right)\right)$$

(2) $$F_{i}({\bar{\rho }}_{i})={AE}_{c}\frac{{\bar{\rho }}_{i}}{{\bar{\rho }}^{0}}In\left(\frac{{\bar{\rho }}_{i}}{{\bar{\rho }}^{0}}\right)$$

where $F_{i}$, ${\bar{\rho }}_{i}$, $\varphi_{ij}$ correspond to the embedding function, the background electron density at site i, and the pairwise interaction between atoms i and j by a distance $R_{ij}$, respectively. $S_{ij}$ denotes the many-body screening function; when $S_{ij}=1$, the interaction between i and j remains unscreened. A is a tunable parameter, $E_{c}$ represents the sublimation energy, and ${\bar{\rho }}^{0}$ refers to the background electron density of the reference structure. This quantity is constructed from the partial electron density contributions associated with distinct angular components [21]:

(3) $${\bar{\rho }}_{i}={\rho_{1}}^{\left(0\right)}G(\Gamma )$$

where

(4) $$G(\Gamma )=\frac{2}{\left(1+e^{-\Gamma }\right)}$$

and

(5) $$\Gamma ={\sum_{h=1}^{3}t_{i}^{\left(h\right)}\left(\frac{\rho_{i}^{\left(h\right)}}{\rho_{i}^{\left(0\right)}}\right)}^{2}$$

Here, $t_{i}^{\left(h\right)}$ represents a set of tunable parameters. The electron density corresponding to individual atoms can be computed via the formula presented below:

(6) $$\rho_{j}^{\left(h\right)}(R)=\rho_{0}e^{-\beta^{\left(h\right)}(\frac{R}{r_{e}}-1)}$$

In Equation (6), $\beta^{\left(h\right)}$ (h = 0, 1, 2, 3) serve as tunable parameters, while $r_{e}$ denotes the nearest-neighbor separation in the equilibrium reference configuration. Additionally, the energy associated with each atom within a specified reference structure can be evaluated by means of the universal Rose equation of state [22]:

(7) $$F[{\bar{\rho }}^{0}(R)]+\frac{1}{2}\sum \phi (R)=E^{u}(R)=-E_{c}(1+\alpha^{*}+\alpha_{3}\alpha^{*3})e^{{-}^{\alpha^{*}}}$$

where

(8) $$\alpha^{*}=\alpha (\frac{R}{r_{e}}-1)$$

and

(9) $$\alpha_{3}=d_{repuls},\;\alpha^{*}<0\;and\;\alpha_{3}=d_{attract},\;\alpha^{*}\geq 0$$

α is a tunable parameter that incorporates contributions from the body of laws, cohesive energy, and equilibrium atomic volume. Also, $d_{repuls}$ and $d_{attract}$ are treated as adjustable parameters.

The electron pair interaction potentials of Al–Al, Al–Mn, Al–Fe, and Al–Cu can be expressed by the above Equations (1)–(9). The Al–Fe–Si potential function is obtained from the mixed potential function [23]. The corresponding MEAM parameters are obtained from a previous study [24] and presented in Table 1.

### 2.2. Structure

**Figure 1:**
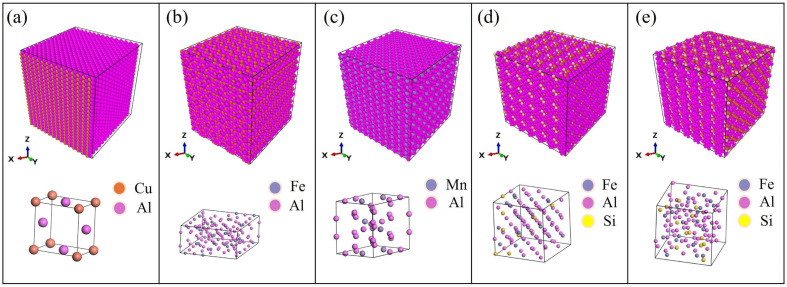
Nanostructures and unit cells of (**a**) Al_2_Cu, (**b**) Al_3_Fe, (**c**) Al_6_Mn, (**d**) Al_8_Fe_2_Si, and (**e**) Al_12_Fe_3_Si_2_.

The nanostructures of Al_6_Mn, Al_2_Cu, and Al_3_Fe are obtained from the unit cells constructed by Barlock et al. [25], Owen et al. [26], and Black et al. [27]. The nanostructures of Al_12_Fe_3_Si_2_ and Al_8_Fe_2_Si are doped in the MS platform according to atomic percentages, and geometrically optimized to obtain stable structures [8,28]. The lattice parameters and dimensions of the structure are shown in Table 2, and the structure and its associated unit cell are depicted in Figure 1.

### 2.3. LAMMPS Environment

Periodic boundary conditions were applied in all three structural directions to eliminate boundary effects arising from the finite-size model. Furthermore, the crystal structures of the IMCs were fully relaxed to ensure they were in equilibrium. Optimized LAMMPS parameters ensured the minimization of the total potential energy and interatomic bonding forces. The conjugate gradient method was used to optimize the structures before applying compressive strain. The structures were relaxed for 10 ps under the canonical ensemble (NVT, constant number of particles, volume, and temperature) to allow the kinetic energy distribution to reach equilibrium, and then were relaxed for 100 ps under the isothermal-isobaric ensemble (NPT, constant number of particles, pressure, and temperature) to ensure that the pressure was balanced with the environment and set at a constant temperature. During simulations, a time step of 0.001 ps was used, and the Nose–Hoover temperature control method was applied for temperature regulation to eliminate internal stresses. Each structure is strained along the Z-Z direction at four strain rates of 1 × 10^10^ s^−1^, 5 × 10^10^ s^−1^, 1 × 10^11^ s^−1^, and 5 × 10^11^ s^−1^, respectively. Stresses in structures originate from the virial stress theorem, as shown in Equation (10) [29]

(10) $$\sigma_{\mathrm{ij}}^{m}=\frac{1}{\Omega^{m}}\left(\frac{1}{2}h^{m}v_{i}^{m}v_{j}^{m}+\sum_{k=1}^{n}r_{mk}^{i}f_{mk}^{j}\right)$$

where m,k: atomic index, $h^{m}$: atomic mass, $v^{m}$: atomic velocity, $r_{mk}$: atomic distance between m and k, $f_{mk}$: atomic force between m and k, $\Omega^{m}$: volume of atomic m.

### 2.4. Method Validation

Previous calculation results are presented in Table 3 to verify the calculation method used in this study. Based on first-principles calculations, extensive studies have been conducted on Al_2_Cu, Al_3_Fe, and Al_6_Mn, including structural, electronic, elastic, and thermodynamic properties, to analyze interatomic interactions, stability, brittleness, etc. [16,30,31,32,33,34,35]. In addition, some experiments have been carried out on IMCs to determine their elastic properties. Tomo et al. [36] evaluated the hardness and Young’s modulus of Al_3_Fe using nanoindentation, while Wang et al. [7] determined the Young’s modulus and final indentation depth of Al_8_Fe_2_Si using nano-hardness testing.

## 3. Results and Discussions

### 3.1. Relaxation Results

**Figure 2:**
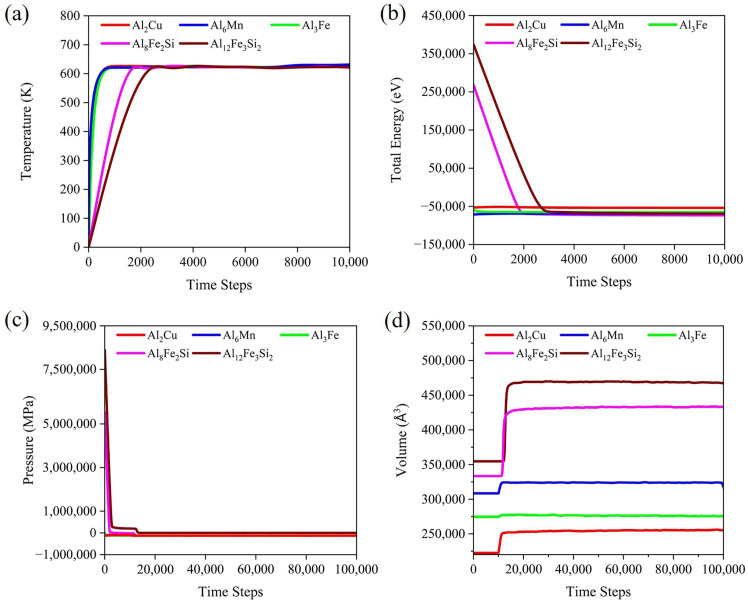
Responses of (**a**) temperature, (**b**) total energy, (**c**) pressure, and (**d**) volume of IMCs during relaxation time.

The single crystal is fully relaxed after the optimization process, combining NVT and NPT. At 623 K, the total energy, temperature, pressure, and volume of the structure during the relaxation period are shown in Figure 2. After applying a combination of NVT cycles with 10,000 time steps (10 ps), the total energy and temperature of the nanostructure clearly tend toward stability, as shown in Figure 2a,b. Due to the structure’s high negative potential energy and low positive kinetic energy, its total energy remains stable in the negative region. This stands in stark contrast to the temperature of 623 K, which is required under specific conditions. Finally, after 100,000 time steps (100 ps) in the NPT ensemble, the nano-structures have stable pressure and volume, as shown in Figure 2c,d. The stable pressure of all structures is 0 MPa. During equilibrium time, the stable volumes of Al_2_Cu, Al_3_Fe, Al_6_Mn, Al_8_Fe_2_Si, and Al_12_Fe_3_Si_2_ IMCs are improved with the increase in pressure in comparison to the initial volume, which is related to the decrease in pressure during the relaxation process.

### 3.2. Stress–Strain Behavior

**Figure 3:**
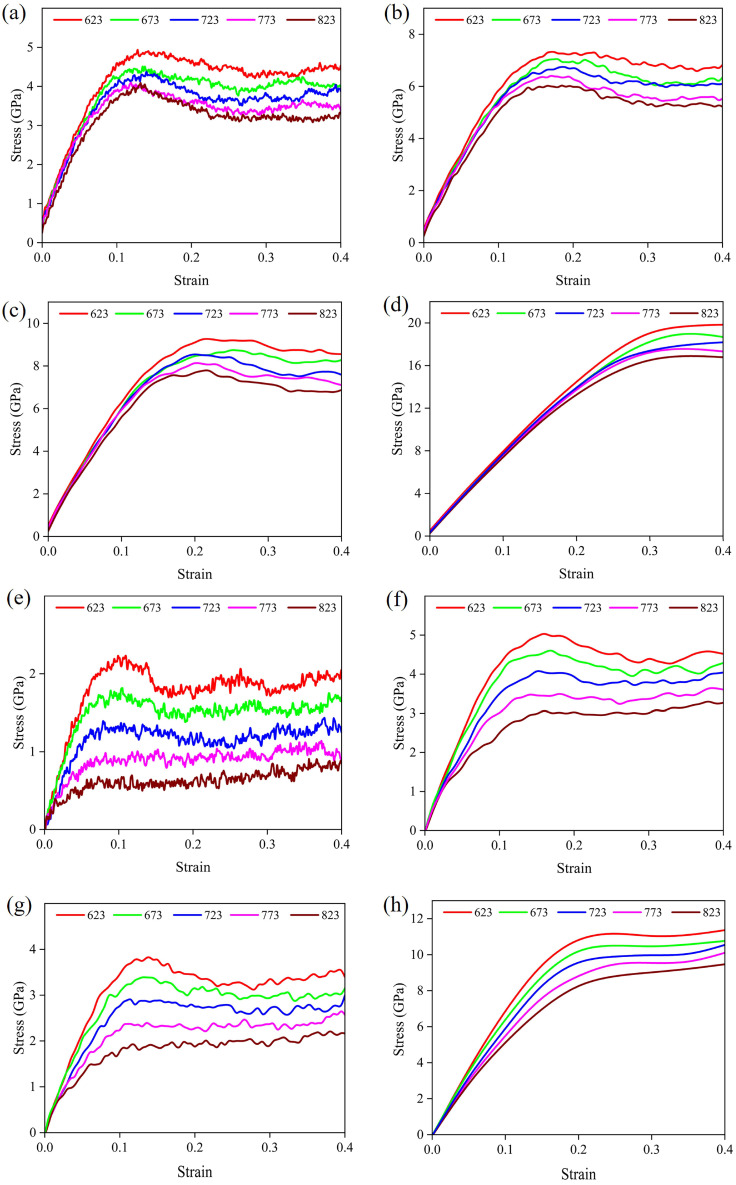

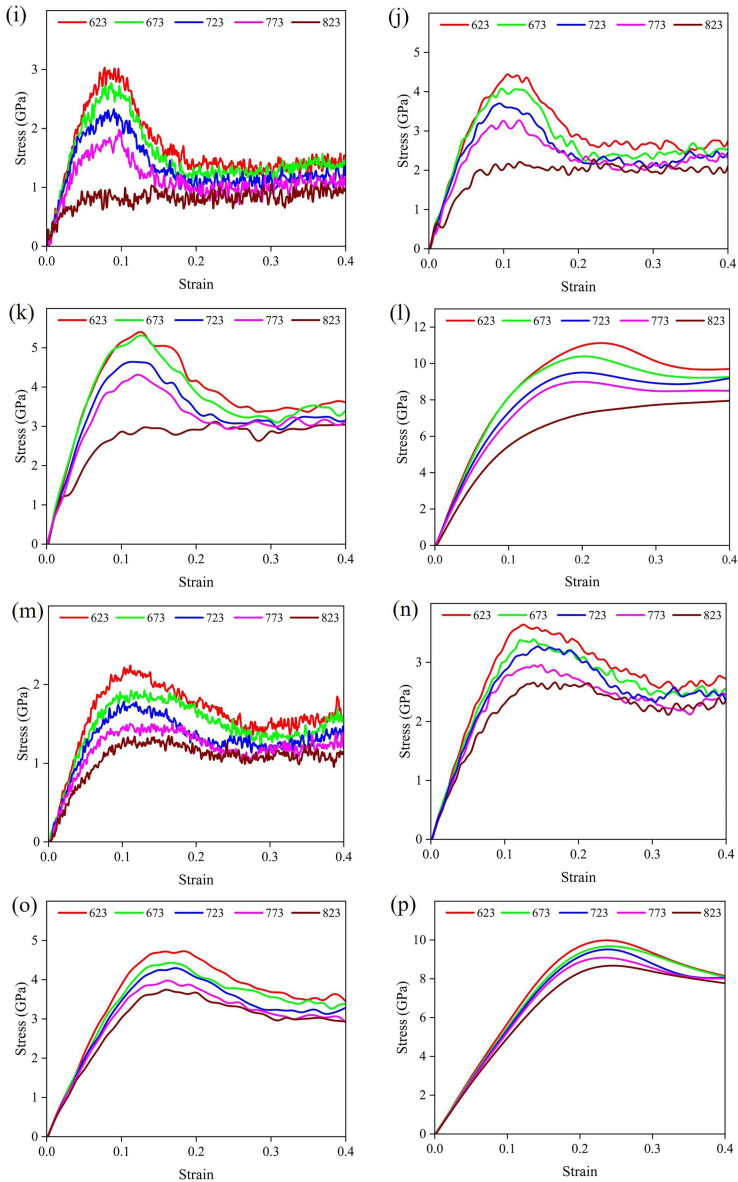

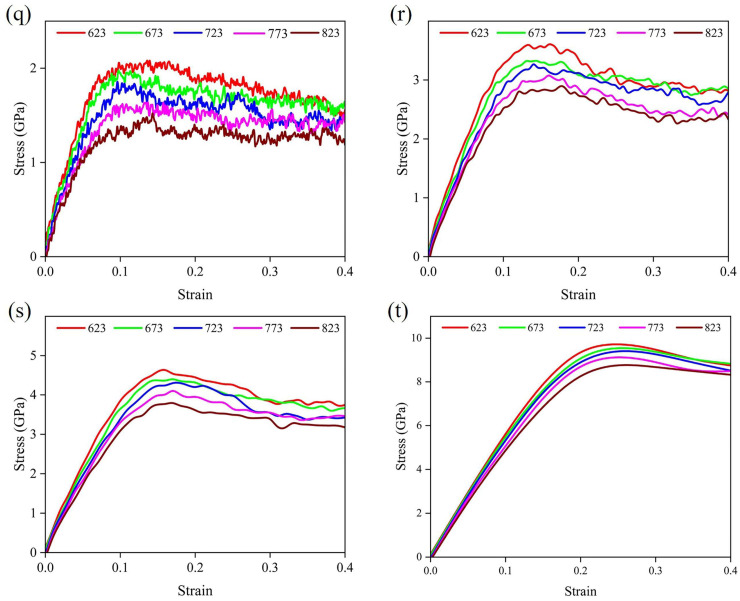
Stress–strain curves of (**a**–**d**) Al_2_Cu, (**e**–**h**) Al_3_Fe, (**i**–**l**) Al_6_Mn, (**m**–**p**) Al_8_Fe_2_Si, and (**q**–**t**) Al_12_Fe_3_Si_2_ at strain rates of 1 × 10^10^ s^−1^, 5 × 10^10^ s^−1^, 1 × 10^11^ s^−1^, and 5 × 10^11^ s^−1^, respectively.

Figure 3 shows the stress–strain curves of the IMCs, from which their compressive properties were derived. These properties exhibit a clear decrease with increasing temperature at a constant strain rate. Moreover, at high strain rates, dislocations within IMCs cannot readily rearrange or annihilate within a short timeframe, leading to increased mutual entanglement and the formation of dense dislocation networks. Consequently, dislocations require greater energy to overcome these obstacles, necessitating higher applied stress to drive deformation. Al_2_Cu displays the highest compressive properties, while Al_12_Fe_3_Si_2_ shows the lowest, as shown in Figure 3a–d,q–t. In contrast, at higher strain rates, the stress–strain curves of IMCs are progressively smoother because the required time for reaching a specific strain is shorter.

**Figure 4:**
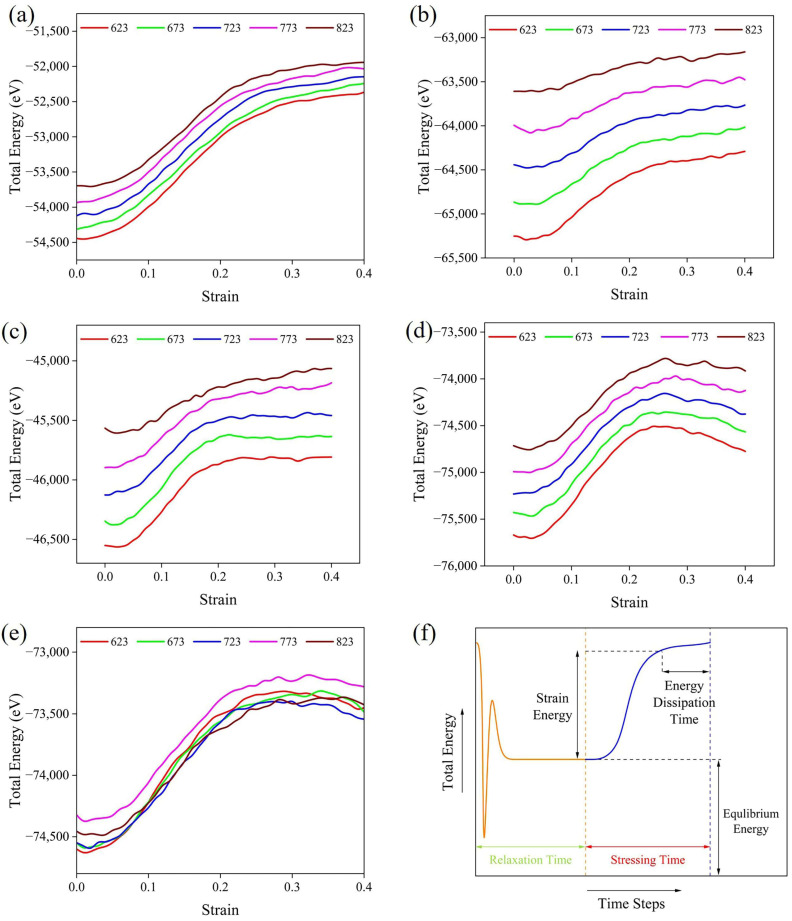
Total energy–strain diagrams of (**a**) Al_2_Cu, (**b**) Al_3_Fe, (**c**) Al_6_Mn, (**d**) Al_8_Fe_2_Si, and (**e**) Al_12_Fe_3_Si_2_ at a strain rate of 1 × 10^11^ s^−1^, and (**f**) curve of total energy–time step.

The deformation behavior of the five IMCs can be divided into linear elastic, non-linear elastic, and non-linear plastic stages. In the linear elastic stage, the stress–strain relationship is directly proportional, as articulated by Hooke’s law [37]. As shown in Figure 4, within the range of strain rates and temperatures in this study, the linear elastic behavior of five IMCs persists up to strains of 0.03~0.149, 0.036~0.149, 0.031~0.099, 0.04~0.149, and 0.047~0.149, respectively. The elastic modulus is the slope of the straight line. At constant temperature, the elastic modulus depends significantly on the strain rate. After reaching a certain strain value, the curve enters the nonlinear elastic stage, showing a nonlinear relationship, and this stage terminates at the yield point. The deformation is reversible before the yield point. In this work, the appropriate strain values equivalent to the yield point of Al_2_Cu, Al_3_Fe, Al_6_Mn, Al_8_Fe_2_Si, and Al_12_Fe_3_Si_2_ are 0.126~0.299, 0.1~0.199, 0.082~0.199, 0.118~0.229, and 0.104~0.249, respectively. After yielding, plastic deformation of the material begins at its ultimate stress point, which is identified as the ultimate compressive strength [38]. It can be seen from Figure 3 that the ultimate compressive strength of Al_2_Cu, Al_3_Fe, Al_6_Mn, Al_8_Fe_2_Si, and Al_12_Fe_3_Si_2_ are 3.9 GPa~19.8 GPa, 0.7 GPa~11.2 GPa, 1.0 GPa~11.2 GPa, 1.3 GPa~10.1 GPa, and 1.1 GPa~9.8 GPa, respectively. It is evident that an elevated strain rate results in enhanced strain and compressive strength. Among the five IMCs, Al_2_Cu exhibits better compressive resistance at high strain rates.

The total energy of the nanostructure varies at different stages of the time step. Once the equilibrium state has been achieved, the crystals are subjected to a constant strain and temperature environment in the simulation. Figure 4 shows the total energy response curves of five IMCs at a strain rate of 1 × 10^11^ s^−1^. Typical curve diagrams with several marked key characteristic points are applied to describe the variation in energy. The simulated system reaches a stable state at the conclusion of the relaxation stage, during which the system energy remains constant over time. In the elastic stage, atoms deviate from their equilibrium positions, and atomic spacing decreases. As a result, bond lengths and bond angles change, leading to an increase in strain energy (mainly elastic energy). At this point, the system energy enhancement is mainly caused by the accumulation of elastic stored energy. When the strain exceeds the elastic limit, part of the strain energy is dissipated due to interatomic bond breakage, defect formation, and slip. Furthermore, the dissipated strain energy is transformed into defect and thermal energy, resulting in a slowdown in the growth rate of total energy. When the strain energy and plastic dissipation are in dynamic equilibrium, the curve remains stable with strain. When the energy of plastic dissipation is higher than strain energy, the total energy decreases, and the curves present a downward trend with strain. The area under the stress–strain curve represents the strain energy absorbed per unit volume of material [39]. Therefore, the Al_2_Cu structure achieves the maximum strain energy before the peak, with a range of 2093 eV to 1751 eV.

### 3.3. Strain Evolution

**Figure 5:**
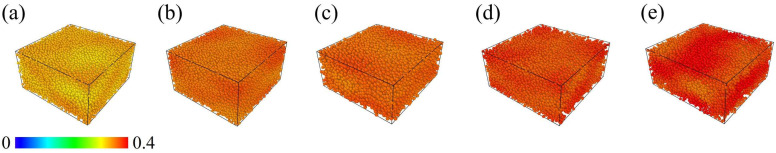
Strain contour plots of (**a**) Al_2_Cu, (**b**) Al_3_Fe, (**c**) Al_6_Mn, (**d**) Al_8_Fe_2_Si, and (**e**) Al_12_Fe_3_Si_2_ at 1 × 10^11^ s^−1^ and 623 K.

The contour plots of shear strain at a strain rate of 1 × 10^11^ s^−1^ and a temperature of 623 K are shown in Figure 5. High shear strain regions typically contain a high density of dislocations and dislocation tangles. These defects significantly disrupt lattice periodicity and weaken interatomic bonding, leading to more pronounced shape changes in the material and a reduction in its elastic modulus [40]. The larger shear strain leads to greater shape change and a smaller elastic modulus of the material. It can be seen that high shear strain is achieved in the Al-Fe-Si system, indicating that the elastic moduli of Al_8_Fe_2_Si and Al_12_Fe_3_Si_2_ are smaller than those of the other three IMCs in this work.

### 3.4. Temperature Effects on Compressive Properties

**Figure 6:**
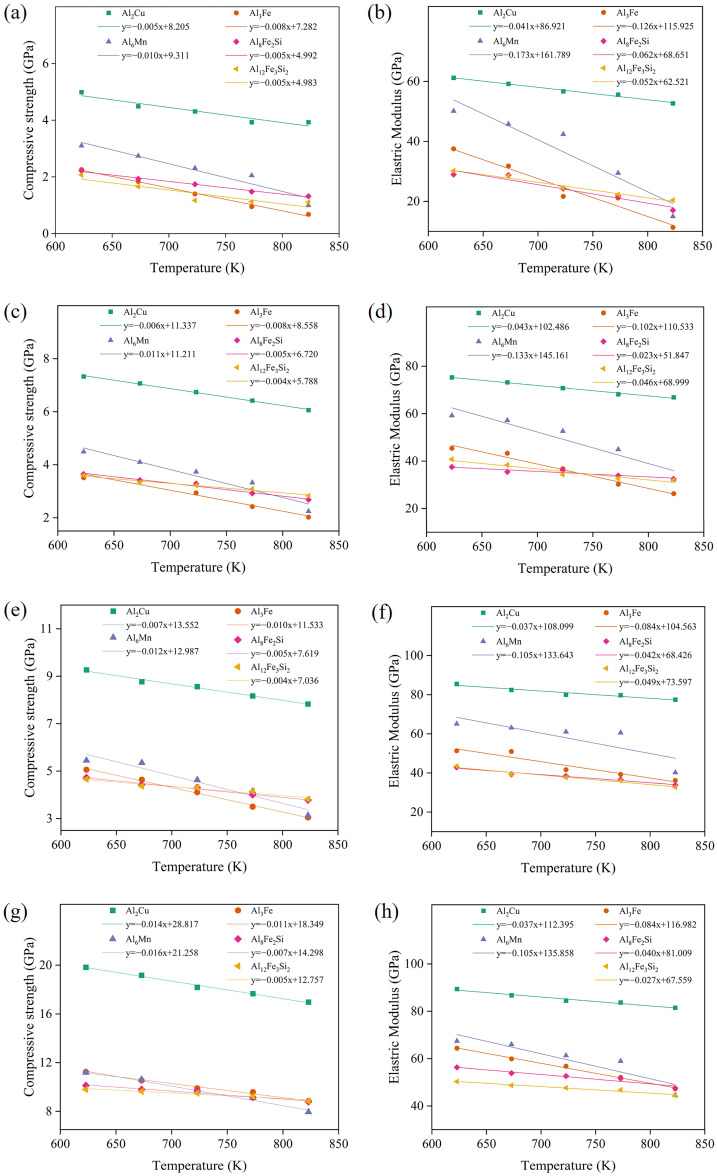
Changes in compressive strength and elastic modulus of IMCs with temperature ranging from 623 K to 823 K and strain rates of (**a**,**b**) 1 × 10^10^ s^−1^, (**c**,**d**) 5 × 10^10^ s^−1^, (**e**,**f**) 1 × 10^11^ s^−1^, and (**g**,**h**) 5 × 10^11^ s^−1^.

The variation in compressive strength and elastic modulus with temperature within the range of 623 K to 823 K is shown in Figure 6. Linear fitting of the experimental data was performed, and the slope of the fitted line can be used to characterize the response of the material’s mechanical properties to temperature changes. This slope value serves as a reference indicator for evaluating the temperature sensitivity of the materials. Due to the decrease in material strength at elevated temperatures, the compressive strengths of all five IMCs exhibit a slight declining trend under high-temperature conditions. At a strain rate of 1 × 10^10^ s^−1^, as the temperature increases from 623 K to 823 K, the slopes of compressive strength for the five IMCs range between 0.05 and 0.10. Among them, Al_6_Mn is the most significantly affected by temperature, while Al_2_Cu is the least affected. At strain rates of 5 × 10^10^ s^−1^, 1 × 10^11^ s^−1^, and 5 × 10^11^ s^−1^, the compressive strength slopes within the same temperature range are 0.004 to 0.011, 0.004 to 0.012, and 0.005 to 0.016, respectively. It is worth noting that among the five IMCs, Al_12_Fe_3_Si_2_ displays the lowest compressive performance, while Al_3_Fe and Al_6_Mn exhibit the highest temperature sensitivity and the poorest stability over the investigated temperature interval.

In the elastic regime of IMCs, the elastic modulus remains constant at specific temperatures and strain rates. When IMCs are subjected to elevated temperatures, the weakening of interatomic bonding leads to a reduction in their elastic capacity, resulting in a decrease in the slope of the stress–strain curve. At a strain rate of 1 × 10^10^ s^−1^, as the temperature rises from 623 K to 823 K, the linear slopes for the five IMCs range from 0.041 to 0.173. Among them, Al_3_Fe and Al_6_Mn show the most pronounced decreasing trends, while Al_2_Cu shows the smallest decrease. At strain rates of 5 × 10^10^ s^−1^, 1 × 10^11^ s^−1^, and 5 × 10^11^ s^−1^, the slopes within the same temperature range are 0.023 to 0.133, 0.037 to 0.105, and 0.027 to 0.105, respectively. It should be noted that Al_2_Cu demonstrates the best stability at higher temperatures, whereas Al_6_Mn exhibits the lowest stability under high-temperature conditions.

### 3.5. The Strain Rate Effect on Compressive Properties

**Figure 7:**
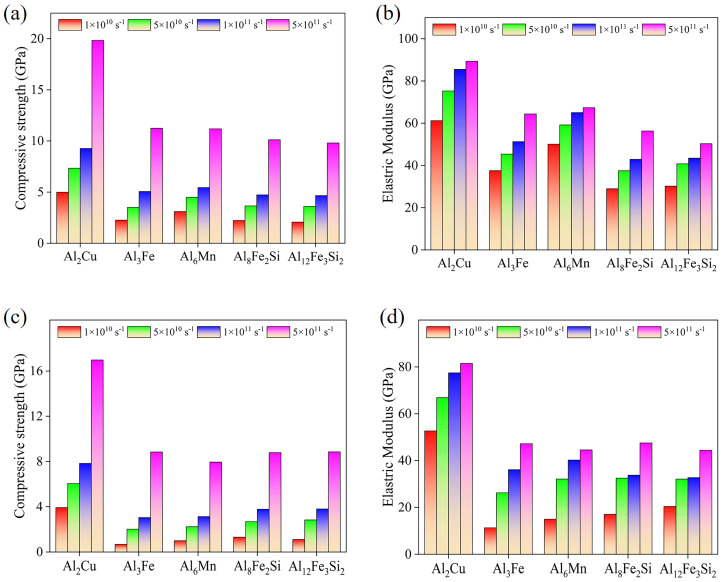
Changes in compressive strength and elastic modulus of IMCs with strain rate at (**a**,**b**) 623 K and (**c**,**d**) 823 K.

Based on the consideration of five different temperatures to observe the properties of IMCs, the effects of strain rate on compressive strength and elastic modulus at the lowest and highest temperatures are described in Figure 7. At each temperature, the compressive strength of these IMCs increases with increasing strain rate. At 623 K, when the strain rate increases from 1 × 10^10^ s^−1^ to 5 × 10^11^ s^−1^, the compressive strengths of Al_2_Cu, Al_3_Fe, Al_6_Mn, Al_8_Fe_2_Si, and Al_12_Fe_3_Si_2_ increase by 74.5%, 79.3%, 72.6%, 78.1%, and 79.0%, and their elastic moduli increase by 31.2%, 41.1%, 25.4%, 48.2%, and 39.2%, respectively. At 823 K, when the strain rate increases from 1 × 10^10^ s^−1^ to 5 × 10^11^ s^−1^, the compressive strengths of Al_2_Cu, Al_3_Fe, Al_6_Mn, Al_8_Fe_2_Si, and Al_12_Fe_3_Si_2_ increase by 76.2%, 92.3%, 87.1%, 84.3%, and 87.1%, and their elastic moduli increase by 35.0%, 76.2%, 66.4%, 64.2%, and 53.3%, respectively. These results suggest that at higher temperatures, the variation in compressive properties such as compressive strength and elastic modulus is wider.

### 3.6. The Strain Rate Sensitivity of Compressive Strength

During the plastic deformation of IMCs, there is an exponential relationship between the applied stress and the strain rate, which can be expressed by the Formula (11) [41]:

(11) $$\sigma =C{\overset{\cdot }{\varepsilon }}^{m}T$$

where σ is the compressive strength, $\overset{\cdot }{\varepsilon }$ is the strain rate, T is the temperature, C is a constant, and m is the strain rate sensitivity coefficient, which is also the hardening index of the compound varying with the strain rate.

When the value of m is above 0, it indicates that the stress of the material increases with an increase in strain rate. When the value of m is below 0, the stress decreases with an increase in strain rate. A higher m value indicates that the material has a stronger ability to deform uniformly when the stamping speed changes, and thus better formability is achieved. Taking the logarithm of the above formula gives the following expressions:

(12) $$\mathrm{In}\sigma =\mathrm{In}(CT)+m\mathrm{In}\overset{\cdot }{\varepsilon }$$

In this experiment, all simulations are conducted under isothermal conditions. The strain rate sensitivity coefficient is based on this condition and is achieved by calculating the slope of Inσ − Inε, which is:

(13) $$m=\frac{\partial \mathrm{In}\sigma }{\partial \mathrm{In}\overset{\cdot }{\varepsilon }}$$

**Figure 8:**
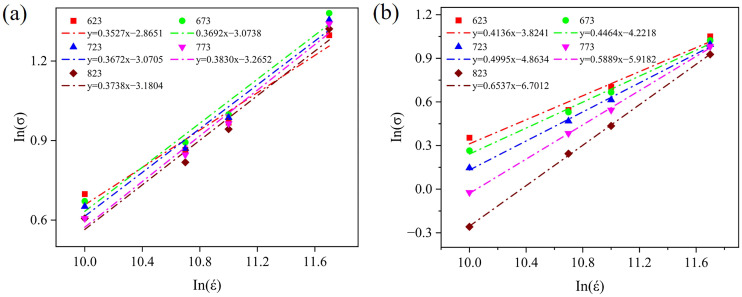

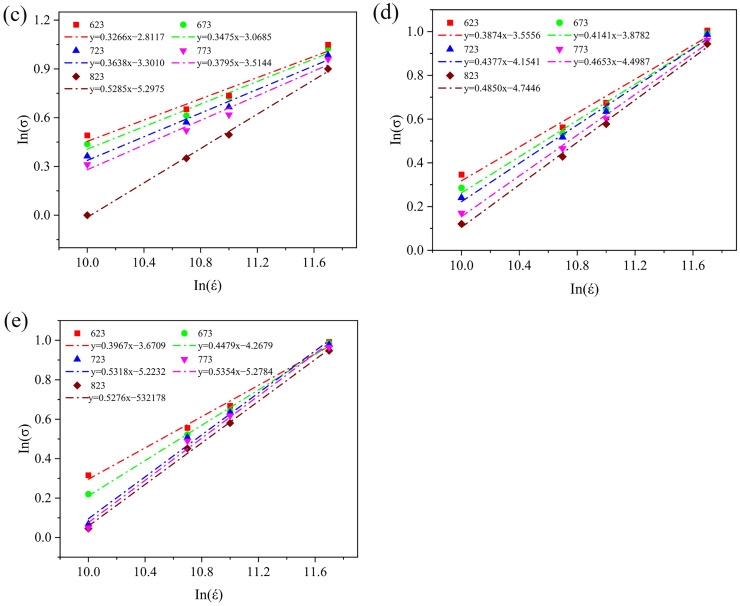
Logarithmic plots of tensile strength vs. strain rate for (**a**) Al_2_Cu, (**b**) Al_3_Fe, (**c**) Al_6_Mn, (**d**) Al_8_Fe_2_Si, and (**e**) Al_12_Fe_3_Si_2_.

Figure 8 shows the Inσ − Inε relationship curves of the five IMCs at 623 K~823 K. Strain rate sensitivity is obtained by substituting the results into the linear equation. The intercept on the Inσ axis can predict the logarithm of strength at a strain rate of 1s^−1^. The results reveal that both strain rate sensitivity and strength are temperature-dependent. As the temperature increases, the strain rate sensitivity of the five IMCs increases. When the temperature rises from 623 K to 823 K, the strain rate sensitivity values of Al_2_Cu, Al_3_Fe, Al_6_Mn, Al_8_Fe_2_Si, and Al_12_Fe_3_Si_2_ range from 0.3527 to 0.3738, 0.4136 to 0.6537, 0.3266 to 0.5285, 0.3874 to 0.4850, and 0.3967 to 0.5276, respectively. Within the temperature range of 623 K to 823 K, the strain rate sensitivities of Al_3_Fe and Al_6_Mn are higher than those of the other three IMCs, and Al_2_Cu exhibits optimal stability throughout the entire temperature range. Notably, though the strain-rate effect weakens at low temperatures, temperature exerts a more significant influence on compressive strength when the strain rate is low.

## 4. Conclusions

This study aims to investigate the stress–strain behavior of intermetallic compounds in 3xxx aluminum alloys as a function of temperature and strain rate. The variation ranges of ultimate compressive strength of Al_2_Cu, Al_3_Fe, Al_6_Mn, Al_8_Fe_2_Si, and Al_12_Fe_3_Si_2_ single-crystal IMCs with temperature and strain rate are displayed as follows: 3.9 GPa~19.8 GPa, 0.7 GPa~11.2 GPa, 1.0 GPa~11.2 GPa, 1.3 GPa~10.1 GPa, and 1.1 GPa~9.8 GPa, respectively. The elastic moduli of the single crystals are 52.7 GPa~89.4 GPa, 11.3 GPa~64.4 GPa, 14.9 GPa~67.4 GPa, 17.1 GPa~56.3 GPa, and 20.4 GPa~50.3 GPa, respectively. Among all IMCs, Al_2_Cu exhibits high mechanical properties and superior stability over a wide range of temperatures and strain rates. while Al_12_Fe_3_Si_2_ shows relatively low compressive strength, and Al_3_Fe and Al_6_Mn display the poorest thermal stability. Furthermore, the compressive strength of the five IMCs displays a high dependence on temperature at lower strain rates but a less dependence on strain rate at lower temperatures. In conclusion, to enhance the stability of 3xxx alloys during thermal processing, it is essential to promote the precipitation and control of the Al_2_Cu phase while maintaining high strength, and strictly restrain the formation of unstable phases such as Al_3_Fe and Al_6_Mn to avoid performance fluctuations due to increased property sensitivity.

## Figures and Tables

**Table 1:** The MEAM parameter.

Element	$E_{c}$	$R_{0}$	α	A	$\beta_{0}$	$\beta_{1}$	$\beta_{2}$	$\beta_{3}$	$t_{1}$	$t_{2}$	$t_{3}$	$\rho_{0}$
Al	3.36	4.05	4.53	0.90	1.89	0.10	6.97	0.007	−0.74	−1.89	9.121	1.0
Cu	3.54	3.62	5.10	0.93	3.75	5.58	5.74	3.14	6.23	2.70	0.87	1.0
Fe	4.29	2.83	5.02	0.43	1.89	0	0.12	0.11	10.74	−4.26	−4.30	1.0
Mn	2.91	2.47	7.87	0.53	8.58	0.001	0.32	4.99	8.28	19.00	−17.34	1.0
Al_2_Cu	3.28	2.45	2.80	--	--	--	--	--	--	--	--	--
Al_3_Fe	3.30	2.59	5.00	--	--	--	--	--	--	--	--	--
Al_6_Mn	3.03	2.28	5.00	--	--	--	--	--	--	--	--	--

**Table 2:** Lattice parameter and structure size.

	Lattice Parameter						Structure Dimension		
IMC	$a(\overset{\circ }{A})$	$b(\overset{\circ }{A})$	$c(\overset{\circ }{A})$	$\alpha {(}^{\circ })$	$\beta {(}^{\circ })$	$\gamma {(}^{\circ })$	$X(\overset{\circ }{A})$	$Y(\overset{\circ }{A})$	$Z(\overset{\circ }{A})$
Al_2_Cu	4.11	4.11	2.88	90.0	90.0	90.0	61.6	61.6	66.5
Al_3_Fe	15.49	8.08	12.47	90.0	107.7	90.0	62.0	64.6	74.8
Al_6_Mn	6.38	7.46	8.76	90.0	90.0	90.0	70.2	67.2	70.1
Al_8_Fe_2_Si	11.78	11.77	11.79	90.0	90.0	90.0	70.7	70.6	70.7
Al_12_Fe_3_Si_2_	11.77	11.81	11.81	90.0	90.0	90.0	70.6	70.8	70.9

**Table 3:** The obtained results are compared with previous literature.

Properties	Previous Experimental Study (0 K–100 K)	From This Study (623 K–823 K)
$E_{{\mathrm{Al}}_{2}\mathrm{Cu}}$	99 GPa~120 GPa [30,31,32,33]	52.68 GPa~89.38 GPa
$E_{{\mathrm{Al}}_{3}\mathrm{Fe}}$	133 GPa~140.9 GPa [34,36]	11.33 GPa~64.39 GPa
$E_{{\mathrm{Al}}_{6}\mathrm{Mn}}$	126.6 GPa [16]	14.94 GPa~67.38 GPa
$B_{{\mathrm{Al}}_{6}\mathrm{Mn}}$	102.6 GPa~121 GPa [35]	
$E_{{\mathrm{Al}}_{8}{\mathrm{Fe}}_{2}\mathrm{Si}}$	148.6 GPa [7]	17.05 GPa~56.31 GPa
$E_{{\mathrm{Al}}_{12}{\mathrm{Fe}}_{3}Si_{2}}$	--	20.42 GPa~50.31 GPa

## Data Availability

The original contributions presented in the study are included in the article. Further inquiries can be directed to the corresponding authors.
